# Pain assessment tools in patients with substance use disorders: a systematic scoping review

**DOI:** 10.1186/s13722-026-00686-y

**Published:** 2026-06-11

**Authors:** Robert W. Hurley, Elizabeth Shilling, Jennifer B. Oliver, Cara L. McDonell, Steven T. Abriola, Meredith C. B. Adams

**Affiliations:** 1 Departments of Anesthesiology, Translational Neuroscience, and Public Health Sciences Pain Outcomes Lab, Wake Forest University School of Medicine, Winston-Salem, NC 27157 USA; 2 Pain Outcomes Lab, Wake Forest University Health Sciences, Winston-Salem, NC USA; 3 Department of Surgery, Wake Forest University School of Medicine, Winston-Salem, NC USA; 4 Department of Anesthesiology, Wake Forest University School of Medicine, Winston-Salem, NC USA; 5 Addiction Medicine, Wake Forest University School of Medicine, Winston-Salem, NC USA; 6 Center for Artificial Intelligence Research, Wake Forest University School of Medicine, Winston-Salem, NC USA

**Keywords:** Pain measurement, Substance use disorders, Opioid use disorder, Chronic pain, Pain interference, Psychometrics, Brief Pain Inventory, Scoping review, NIH HEAL Initiative, Multidimensional pain assessment

## Abstract

**Background:**

Chronic pain and substance use disorders (SUD) frequently co-occur and are associated with poorer treatment outcomes. The way pain is measured affects which patients are identified as having clinically meaningful pain, which functional consequences are linked to substance use outcomes, and whether findings can be compared across studies or translated into care. Pain measurement practices in SUD research have not been systematically mapped.

**Methods:**

We conducted a systematic scoping review of studies from 1990 to 2025 that reported pain assessment in SUD populations, including opioid, alcohol, tobacco, stimulant, and polysubstance use disorders. We searched PubMed, MEDLINE, Embase, CINAHL and PsycINFO. We extracted data on pain assessment tools, substance use categories, SUD-specific psychometric evaluation, and coverage of eight NIH HEAL Pain Common Data Element domains: intensity, interference, location, physical functioning, quality of life, psychological factors, sleep disturbance, and treatment satisfaction.

**Results:**

Ninety-six studies (25,872 participants; mean per study 269, median 113) met inclusion criteria. Most used unidimensional pain intensity measures. Pain intensity scales appeared in 91% of studies, but only 14.6% reported SUD-specific psychometric evaluation. Pain interference, location, and treatment satisfaction were assessed in fewer than 60% of studies, and 11.5% assessed at least six of the eight NIH HEAL domains. Randomized controlled trials assessed more domains on average than cross-sectional studies. Studies focusing on opioid use disorder (OUD) more often used multidimensional tools than studies of alcohol, tobacco, stimulant, or polysubstance use. In the subset of studies that examined pain-substance use relationships, pain interference more often showed stronger associations with substance use outcomes than pain intensity alone.

**Conclusions:**

Pain assessment in SUD research relies largely on unidimensional intensity scales. Substance Use Disorder specific validation is infrequent, and multidimensional pain domains are not consistently used. Brief multidimensional assessment approaches and systematic psychometric evaluation of pain measures in SUD populations are needed to improve the accuracy and clinical usefulness of pain measurement in research and treatment. We propose a candidate framework and minimum set for such studies.

**Supplementary Information:**

The online version contains supplementary material available at https://doi.org/10.1186/s13722-026-00686-y.

## Background

Chronic pain and substance use disorders (SUD) frequently co-occur. They contribute to complex clinical presentations and poorer outcomes across medical and addiction settings [1–3]. Chronic pain can increase the risk of SUD through self-medication and maladaptive coping. Ongoing substance use can in turn exacerbate pain sensitivity, interfere with treatment, and worsen functional and quality of life outcomes [4–7]. Untreated or undertreated pain in SUD populations has been associated with relapse, reduced treatment retention, and diminished quality of life. Accurate and clinically meaningful pain assessment is therefore needed in this population [6–8]. 

Pain assessment in SUD populations presents specific challenges that tools developed for general chronic pain populations may not fully address [9–14]. Under the International Association for the Study of Pain (IASP) definition, pain is an unpleasant sensory and emotional experience regardless of underlying mechanism. Withdrawal-related somatic distress therefore constitutes a valid pain experience rather than a non-pain confound. The more precise measurement issue is one of construct heterogeneity. A single self-reported pain rating in an SUD population may reflect several co-occurring contributors that vary across time and treatment state. These contributors differentially shape how measurement tools, particularly unidimensional intensity scales, should be interpreted. Clinician concerns about medication-seeking, the well-recognized coexistence of long-term opioid therapy for chronic pain and opioid use disorder (OUD), the coexistence of which complicates rather than precludes accurate diagnosis, and stigma toward people with SUD can all add to the challenges of pain assessment and contribute to undertreatment [15–20]. In this context, both under- and over-attribution of symptoms to specific pain processes are possible, with implications for diagnosis, treatment planning, and patient trust.

Pain ratings in SUD populations can be understood as arising from five interacting contributors that current measurement tools capture unevenly. The first is sensory or nociceptive input from peripheral injury or disease. The second is nociplastic mechanisms, including SUD-related central nervous system pain sensitization. The third is withdrawal-related somatic distress, whose neural substrates overlap substantially with those of hyperkatifeia and nociplastic pain [21–23]. The fourth is affective and cognitive processes such as negative affect, catastrophizing, and craving. The fifth is functional consequences, often captured as pain interference. Framing the measurement problem in terms of these contributors clarifies what each tool captures and what it leaves out, and it provides the organizing structure for the synthesis that follows.

Despite growing recognition of the importance of pain in SUD treatment, pain measurement practices in SUD populations have not been systematically mapped. It is not well described which pain tools are used in SUD research, to what extent those tools have been psychometrically evaluated in SUD samples, how well existing assessments cover multidimensional pain domains, or how these patterns vary across substances, settings, and study designs. The extent to which current measurement practices reflect evolving standards, such as increasing attention to pain interference and functioning, also remains unclear.

To address these gaps, we conducted a systematic scoping review of pain assessment in SUD research from 1990 to 2025, following established scoping review guidelines [24, 25]. The 1990 start date brackets the development and dissemination of the first generation of validated multidimensional pain measures, including the Wisconsin Brief Pain Questionnaire and its successor, the Brief Pain Inventory (BPI) [11, 26]. Our objectives were to: (1) identify and characterize pain assessment tools currently used across different SUD populations and study contexts; (2) evaluate their validation status specifically for SUD populations; (3) assess coverage of multidimensional pain domains recommended by the NIH HEAL initiative [27–29]; and (4) examine substance-specific considerations in pain assessment. Based on these findings, we also explore measurement implications for SUD care and research. These implications are intended as evidence-informed proposals rather than prescriptive guidelines and will require prospective validation.

## Methods

### Study design and registration

We conducted a systematic scoping review following the Preferred Reporting Items for Systematic Reviews and Meta-Analyses extension for Scoping Reviews (PRISMA-ScR) and the PRISMA search extension (PRISMA-S) [24, 25]. The protocol was registered as a scoping review with OSF (osf.io/7rb83) on January 21, 2025. All review procedures were managed using Covidence systematic review software for systematic documentation and reproducibility [30]. 

### Data sources and search strategy

We searched PubMed, Ovid MEDLINE, Embase, and CINAHL/PsycINFO from January 1, 1990 to July 1, 2025 [11, 26]. A medical research librarian reviewed and refined the search strategy for completeness and specificity. Search strategies combined controlled vocabulary and keywords for pain measurement and substance-related disorders, including terms such as “pain assessment,” “pain measurement,” “pain scale,” “pain questionnaire,” “substance use disorder,” “opioid use disorder,” and “addiction,” with database-specific adaptations. Medical Subject Headings (MeSH) and equivalent controlled vocabulary terms (e.g., “Pain Measurement,” “Pain Assessment,” “Substance-Related Disorders,” “Opioid-Related Disorders”) were used where applicable. Reference lists of included studies and relevant reviews were hand-searched for additional articles. Full search strategies are provided in Supplement 1.

### Eligibility criteria

Inclusion criteria were: (1) original, peer-reviewed research; (2) studies including participants with any diagnosed SUD or receiving SUD treatment; (3) use of at least one formal pain assessment tool or scale with sufficient detail to characterize pain measurement; and (4) publication in English or with an available English translation. We included a range of study designs (e.g., randomized controlled trials, cohort studies, cross-sectional studies, longitudinal observational studies, mixed-methods studies) provided they reported pain assessment methods in SUD populations.

Exclusion criteria were: (1) studies without SUD participants; (2) absence of formal pain assessment (e.g., narrative mention of pain without use of a defined tool or item); (3) conference abstracts, letters, commentaries, or reviews without original data; (4) duplicate reports of the same dataset; and (5) studies focused solely on pain treatment without describing pain assessment methodology.

### Study selection

**Fig. 1:**
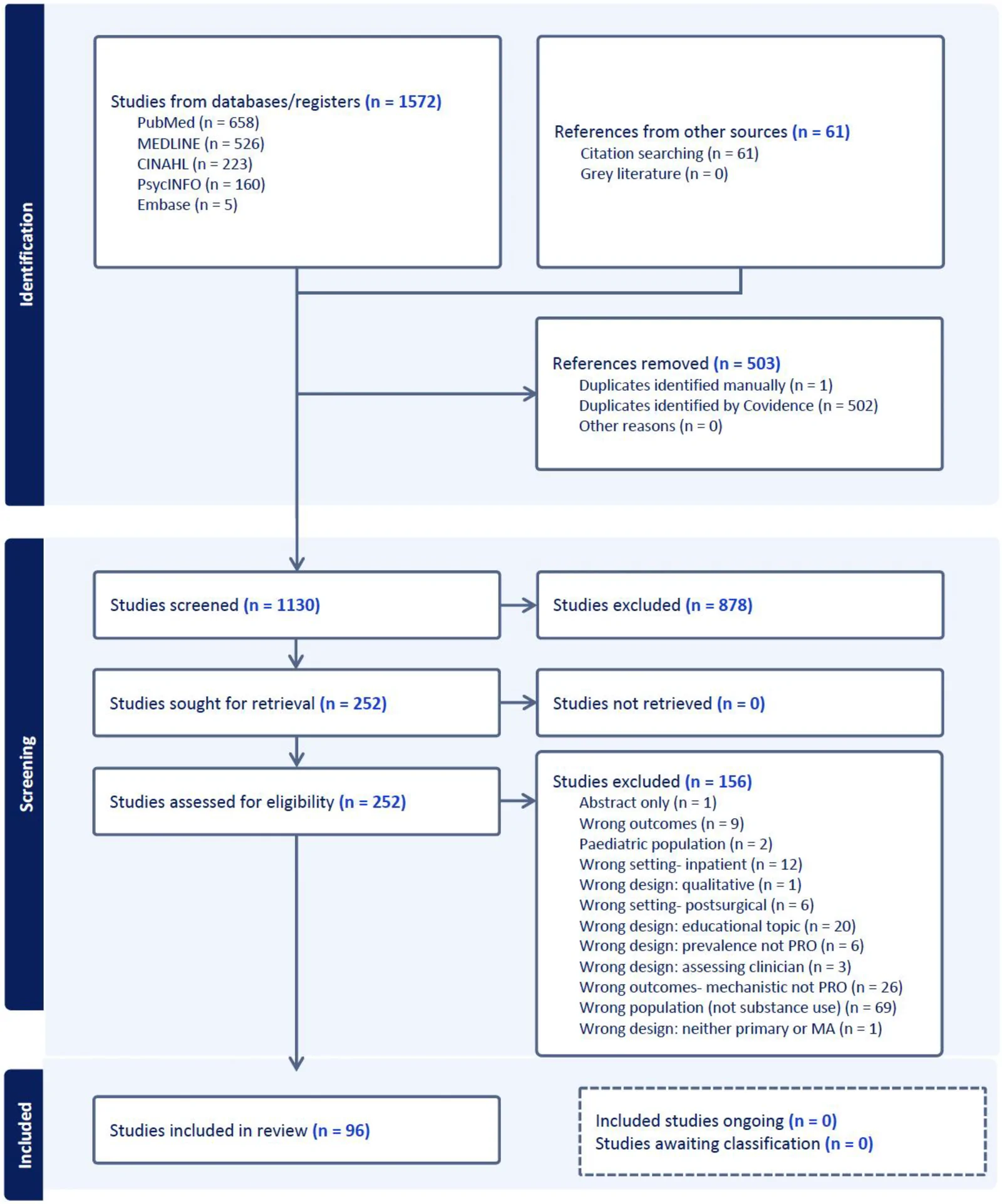
PRISMA flow diagram of study selection process

After removal of duplicates in Covidence, titles and abstracts were screened independently by two reviewers to identify potentially eligible studies. Full-text articles for all potentially eligible records were then reviewed independently by two reviewers (MCBA, RWH) against the inclusion and exclusion criteria. Discrepancies at either stage were resolved through discussion, with a third reviewer (CM) adjudicating when consensus among the team (SA, ES, JO) could not be reached. Reasons for full-text exclusion were recorded in Covidence. The study selection process is summarized in a PRISMA flow diagram (Fig. 1).

### Data extraction and quality assessment

We developed a structured data extraction form to capture study identification (author, year, country), design and setting, sample size and key demographic characteristics, primary SUD type(s), pain assessment tools used (including individual items and multi-item instruments), and whether the tools were explicitly reported as validated or psychometrically evaluated in SUD samples. We define three measurement terms and apply them consistently throughout the manuscript. “Unidimensional” refers to measures that assess a single pain construct, most commonly intensity. “Intensity-only” refers to measures that assess the intensity construct regardless of item count. “Single-item” refers to instruments composed of one item, which are typically but not always intensity-only. “Validated in SUD” denotes any formal psychometric evaluation in an SUD sample (e.g., internal consistency, factor structure, construct or convergent validity, responsiveness) but does not require a complete validation program. Pain assessment content was mapped to 8 NIH HEAL pain CDE domains, a federally coordinated consensus set of domains intended to operationalize a biopsychosocial model of chronic pain and enable harmonized measurement across HEAL-funded studies: intensity, interference, location, physical functioning, quality of life, psychological factors, sleep disturbance, and treatment satisfaction [1, 27–29, 31]. These domains were articulated after many included studies were conducted but were selected a priori as a consistent framework for synthesis.

Two reviewers (RWH, MCBA) independently extracted data from each included study, and a third reviewer (CM) checked all entries for accuracy and completeness. Discrepancies were resolved by consensus. In addition to the primary extraction domains, we recorded substance-specific assessment patterns (e.g., differences in tools used for opioid versus alcohol or stimulant use disorders) and, as a secondary descriptive dimension, whether studies reported associations between pain measures and substance use-related outcomes (e.g., relapse, treatment retention, substance use severity).

### Methodological quality assessment

Because our primary goal was to map pain measurement approaches rather than estimate pooled effects, formal risk-of-bias assessment was not used to exclude studies. To provide context for the evidence base, we evaluated methodological quality using a modified Newcastle-Ottawa Scale adapted for pain assessment research in SUD populations [32, 33]. The scale assessed Selection (0–4 points), Comparability (0–2 points), and Outcome (0–3 points) domains, with higher scores indicating higher methodological quality. Two independent reviewers (MCBA, RWH) rated each study, and disagreements were resolved through discussion (SA, ES, JO). Quality scores were summarized descriptively by study design and were not used as weights in synthesis.

### Data Synthesis

Given the heterogeneity of study designs, populations, and outcomes, we conducted a narrative synthesis consistent with scoping review methodology and did not perform meta-analysis. We organized the synthesis around: (1) types and frequencies of pain assessment tools used; (2) reporting of SUD-specific validation or psychometric evaluation; (3) coverage of NIH HEAL pain domains; and (4) substance-specific assessment patterns. To account for changes in clinical practice and measurement standards over time, we additionally described patterns by publication period (e.g., early vs. later years) in sensitivity analyses. Exploratory observations on associations between pain measures and substance use outcomes were summarized qualitatively. We did so to illustrate how different measurement approaches may relate to clinically relevant endpoints, while acknowledging that these were not primary predefined outcomes of the scoping review.

## Results

### Study selection and characteristics

The search yielded 1,254 records after removal of duplicates, of which 96 studies met inclusion criteria after title/abstract and full-text screening (Fig. 1) [21, 34–128]. Characteristics of the included studies are summarized in Table 1. Studies included 25,872 participants (mean per study 269, median 113, interquartile range 56 to 250). The right-skewed distribution was driven by a small number of large cohort and registry studies. Studies were published between 1990 and 2025, with 4.2% published between 1990 and 2000, 12.5% between 2001 and 2010, 15.6% between 2011 and 2015, 39.6% between 2016 and 2020, and 28.1% between 2021 and 2025 (Table 1). Most studies were conducted in the United States (76.0%), followed by Europe (12.5%), Australia (6.3%), and other regions (5.2%). Sample sizes were predominantly medium-sized (50 to 200 participants; 54.2%), with 16.7% classified as small (< 50) and 29.1% as large (> 200) (Table 1). Outpatient SUD treatment settings were most common (60.4%), followed by inpatient or residential SUD treatment (22.9%), mixed settings (11.5%), and community samples (5.2%) (Table 1).

**Table 1:** Characteristics of included studies (*N* = 96)

Characteristic*	Number	Percentage (%)
**Publication Year**		
1990–2000	4	4.2
2001–2010	12	12.5
2011–2015	15	15.6
2016–2020	38	39.6
2021–2025	27	28.1
**Geographic Region**		
United States	73	76.0
Europe	12	12.5
Australia	6	6.3
Other	5	5.2
**Sample Size**		
Small (< 50 participants)	16	16.7
Medium (50–200 participants)	52	54.2
Large (> 200 participants)	28	29.1
**Study Setting**		
Outpatient SUD treatment	58	60.4
Inpatient/residential SUD Treatment	22	22.9
Mixed settings	11	11.5
Community sample	5	5.2

*Demographic data was inconsistently reported across studies

### Study design and methodological quality

Study design distribution and methodological quality are presented in Table 2. Cross-sectional designs accounted for 52.0% (*n* = 50) of studies, randomized controlled trials for 19.0% (*n* = 18), cohort studies for 12.5% (*n* = 12), longitudinal observational studies for 3.1% (*n* = 3), and other designs (including pilot studies, secondary analyses, mixed-methods, and case series) for 13.5% (*n* = 13) (Table 2). The mean modified Newcastle-Ottawa Scale score was 6.7 out of 9 (range 4 to 9). Randomized controlled trials had higher average quality (mean 7.8) than cross-sectional studies (mean 6.2) (Table 2). Common methodological limitations included inadequate sample size justification (64% of studies), limited control for confounding variables (52%), incomplete reporting of non-respondent characteristics (41%), lack of blinded outcome assessment (38%), and inadequate follow-up periods for pain assessment (33%) (Table 2).

**Table 2:** Study design distribution and quality assessment scores

Study Design	Number	Percentage (%)	Mean NOS* Score	Score Range
Cross-sectional	50	52.0	6.2	4–8
Randomized controlled trials	18	19.0	7.8	6–9
Cohort studies	12	12.5	7.1	5–9
Longitudinal observational	3	3.1	7.4	6–8
Other designs**	13	13.5	5.9	4–7
**Total**	**96**	**100**	**6.7**	**4–9**

*NOS = Modified Newcastle-Ottawa Scale (maximum score = 9)

**Includes pilot studies, secondary analyses, mixed methods, and case series

**Common methodological limitations**:

- Inadequate sample size justification (64% of studies).
- Limited control for confounding variables (52% of studies).
- Incomplete reporting of non-respondent characteristics (41% of studies).
- Lack of blinded outcome assessment (38% of studies).
- Inadequate follow-up period for pain assessment (33% of studies).

### Participant and substance use characteristics

Opioid use disorders were reported in 65 studies (67.7%). These included 27 studies (28.1%) involving methadone maintenance, 15 (15.6%) involving buprenorphine maintenance, and 23 (24.0%) involving other opioid treatment contexts (Table 3). Alcohol use disorders appeared in 21 studies (21.9%), tobacco use in 13 (13.5%), stimulant use disorders in 9 (9.4%), polysubstance use in 26 (27.1%), and other substance types in 7 (7.3%). Many studies included multiple substance types. Substance-specific analyses of pain assessment were less common: 14 OUD studies, 3 alcohol use disorder studies, 8 tobacco studies, 1 stimulant study, and 8 polysubstance studies conducted substance-specific analyses or explicitly addressed substance-specific pain assessment considerations (Table 3).

**Table 3:** Substance use disorder types represented

Substance Type	Number	Percentage (%)	Substance-Specific Pain Assessment*
**Opioid Use Disorders**	**65**	**67.7**	**14**
Methadone maintenance	27	28.1	8
Buprenorphine maintenance	15	15.6	5
Other opioid treatment	23	24.0	1
**Alcohol Use Disorders**	**21**	**21.9**	**3**
**Tobacco Use**	**13**	**13.5**	**8**
**Stimulant Use Disorders**	**9**	**9.4**	**1**
**Polysubstance Use**	**26**	**27.1**	**8**
**Other Substance Types**	**7**	**7.3**	**0**

*Number of studies that conducted substance-specific analyses of pain assessment measures or addressed unique substance-specific considerations in pain assessment methodology

Note: Percentages sum to > 100% as many studies included multiple substance types

### Pain assessment tools used

Across the 96 studies, pain intensity was reported alone or in combination with other domains in 91% (*n* = 87) of studies. The Brief Pain Inventory (BPI) was the most frequently used multidimensional measure, appearing in 22 studies (22.9%), with 6 reporting psychometric evaluation in SUD populations (Table 4). Intensity-only assessment using either a Numeric Rating Scale (NRS) or Visual Analog Scale (VAS) was reported in 6 (6.3%) and 5 (5.2%) studies, respectively, with SUD-specific psychometric evaluation noted in 2 NRS and 1 VAS study (Table 4). The McGill Pain Questionnaire and SF-36 pain components were each used in 3 studies (3.1%), with SUD-specific psychometric evaluation reported for 1 SF-36 application.

Two studies (2.1%) used the Pain Catastrophizing Scale, and 18 (18.8%) used study-specific pain questionnaires. Twelve studies (12.5%) used a single-item pain presence question for screening; in all but one of these, the single-item question was intensity-only. Twenty-five studies (26.0%) used other instruments, including specialized or condition-specific measures. The average number of NIH HEAL pain domains captured varied across tools. The BPI covered the largest number of domains on average, followed by the SF-36 pain components and study-specific multidimensional questionnaires (Table 4).

**Table 4:** Pain assessment tools and domain coverage

Pain Assessment Tool	Number	Percentage (%)	Validated in SUD*	Most Common Usage
Brief Pain Inventory (BPI)	22	22.9	6	Comprehensive pain assessment
Numeric Rating Scale (NRS)	6	6.3	2	Pain intensity only
Visual Analog Scale (VAS)	5	5.2	1	Pain intensity only
McGill Pain Questionnaire	3	3.1	0	Pain quality assessment
SF-36 (pain components)	3	3.1	1	Quality of life studies
Pain Catastrophizing Scale	2	2.1	0	Psychological components
Study-specific questionnaires	18	18.8	2	Varied applications
Single-item pain presence	12	12.5	0	Screening for pain
Other instruments	25	26.0	2	Specialized applications

*Number of studies reporting validation data for the tool in SUD populations

### Validation of pain measures in SUD populations

Substance use disorder-specific psychometric evaluation of pain measures was infrequently reported. Fourteen studies (14.6%) reported psychometric data, most commonly internal consistency, factor structure, or construct validity, for at least one pain measure in SUD samples. Most of this work involved the BPI in OUD populations (Table 4). Few studies described responsiveness or measurement invariance across treatment phase. Evidence for other tools and for non-opioid substance types (e.g., alcohol, stimulants, polysubstance use) was limited and typically restricted to single studies or settings (Table 4). As noted in Table 4 and reiterated in the Methods, “Validated in SUD” in our extraction denotes any formal psychometric evaluation in an SUD sample, not a complete validation program.

### Coverage of NIH HEAL pain domains

Pain intensity was assessed in 72 studies (75.0%), with higher coverage in intervention studies (94.4%) than in cross-sectional studies (86.0%). Pain interference was assessed in 35 studies (36.5%), including 32.0% of cross-sectional and 72.2% of intervention studies. Pain location was reported in 32 studies (33.3%), physical functioning in 8 (8.3%), quality of life in 9 (9.4%), depression in 8 (8.3%), anxiety in 1 (1.0%), sleep disturbance in 1 (1.0%), and global satisfaction with treatment in 1 (1.0%) (Table 5).

**Table 5:** NIH HEAL pain common data elements domain coverage

Pain Domain*	Number	Percentage (%)	Cross-Sectional Studies (%)	Intervention Studies (%)
Pain intensity	72	75.0	86.0	94.4
Pain interference	35	36.5	32.0	72.2
Pain location	32	33.3	28.0	55.5
Physical functioning	8	8.3	6.0	22.2
Quality of life	9	9.4	6.0	27.8
Depression	8	8.3	8.0	16.7
Anxiety	1	1.0	0.0	5.6
Sleep disturbance	1	1.0	0.0	5.6
Global satisfaction with treatment	1	1.0	0.0	5.6
**Studies assessing ≥ 6 domains**	**11**	**11.5**	**4.0**	**27.8**
**Studies assessing ≤ 3 domains**	**52**	**54.2**	**66.0**	**22.2**

*Domain Coverage by Substance Type:

- OUD studies average 2.9 domains.
- AUD studies average 3.2 domains.
- Tobacco studies average 2.2 domains.
- Stimulant studies average 1.3 domains.
- Polysubstance studies average 2.7 domains.

Overall, 11 studies (11.5%) assessed at least six of the eight HEAL pain domains, while 52 (54.2%) assessed three or fewer domains. Cross-sectional studies were more likely to assess three or fewer domains (66.0%), whereas intervention studies were more likely to assess six or more domains (27.8%). Domain coverage also varied by substance type. Opioid use disorder studies assessed an average of 2.9 domains, alcohol use disorder studies 3.2, tobacco studies 2.2, stimulant studies 1.3, and polysubstance studies 2.7 (Table 5).

### Substance-specific assessment patterns

Patterns of pain assessment differed by substance type (Tables 3 and 5). Opioid use disorder focused studies more frequently incorporated multidimensional tools, including the BPI, and had higher average domain coverage than studies focused on other substances. Alcohol use disorder studies more often included psychological domains, such as depression or pain catastrophizing, alongside pain measures. Tobacco-related studies frequently examined links between pain and smoking motivations or behaviors, often using targeted questionnaires that connected pain experiences to tobacco use (Table 3). Stimulant use disorder studies most often relied on single-item or unidimensional intensity assessments, with the lowest average HEAL domain coverage (Table 5).

### Exploratory observations on pain-substance use outcome associations

Although pain-substance use outcome associations were not primary predefined outcomes of this scoping review, many studies reported such relationships. Across studies that examined chronic pain in relation to SUD outcomes, 72% reported associations between chronic pain and less favorable treatment outcomes, including higher relapse rates, shorter abstinence duration, or reduced treatment adherence. In the subset of studies that directly compared pain interference and pain intensity as predictors, pain interference more often showed stronger associations with substance use-related outcomes than intensity alone. Several longitudinal studies reported that changes in pain scores, particularly in interference or functioning domains, were associated with subsequent changes in substance use behaviors, although tools, domains, and follow-up intervals varied. These exploratory observations suggest that multidimensional pain assessment, including interference and functioning, may be more closely aligned with clinically meaningful SUD outcomes than intensity measures alone. Formal evaluation of this hypothesis was beyond the scope of this scoping review.

## Discussion

This systematic scoping review of 96 studies involving 25,872 participants characterizes how pain has been measured in SUD research over the three decades since the BPI was published. We focused on tools used, SUD-specific psychometric evaluation, and coverage of multidimensional pain domains. Most studies used unidimensional pain intensity measures. SUD-specific psychometric evaluation was infrequently reported. Coverage of NIH HEAL pain domains was limited, particularly for pain interference, physical functioning, sleep, and treatment satisfaction. Domain coverage and use of multidimensional tools were somewhat higher in randomized and intervention studies and in OUD-focused work, but few studies assessed six or more domains. In studies that directly compared pain dimensions as correlates of substance use outcomes, pain interference more consistently outperformed pain intensity alone. We discuss this finding in a dedicated subsection below. Several opportunities exist to improve the validity, comparability, and clinical usefulness of pain assessment in SUD populations.

### Measurement gaps and implications for tool selection

The predominance of intensity-only measures, often single-item scales or brief numeric ratings, reflects an implicit sensory model of pain. This model is poorly aligned with contemporary biopsychosocial frameworks and with the inherently behavioral nature of SUD outcomes such as relapse, retention, and functioning. Limited assessment of interference, functioning, psychological comorbidities, and sleep means that many studies, and the clinical programs informed by them, may systematically underestimate the impact of pain on daily life, treatment engagement, and recovery. The small number of studies that assessed six or more HEAL pain domains, combined with low rates of SUD-specific psychometric evaluation, indicates that multidimensional, psychometrically robust measurement has not yet been integrated into SUD clinical research.

The variation in domain coverage across substance type points to the need for deliberate tool selection. OUD studies more frequently used multidimensional instruments such as the BPI and captured more domains on average than studies focused on tobacco, stimulants, or polysubstance use, where intensity-only assessments were common and multidomain coverage was lowest. Alcohol use disorder studies more often incorporated psychological constructs related to pain, such as depression or catastrophizing, but did not consistently assess functioning or treatment satisfaction. These patterns suggest that current measurement practices may differentially capture aspects of pain experience across substance types, complicating cross-study comparisons and potentially obscuring important clinical differences.

### Pain interference as a clinically meaningful target

In studies that directly compared pain dimensions as correlates of substance use outcomes, pain interference more often demonstrated stronger associations than pain intensity alone [64, 89, 129, 130]. This pattern is consistent with the broader pain literature [131–133]. Because interference integrates sensory input, affective response, and functional impact, it tracks behavior-relevant outcomes such as work, mobility, and social participation more reliably than sensory intensity alone [131, 133, 134]. In SUD specifically, this matters. Outcomes that drive treatment success (treatment retention, relapse, return to work and roles) are inherently behavioral [51, 135, 136]. Measurement that captures behavior-relevant pain impact is therefore better aligned with the endpoints of interest than measurement of sensory intensity in isolation [64, 137]. 

For future study design, this implies that interference and functioning warrant treatment as primary or co-primary endpoints rather than as secondary descriptors. This is particularly true in studies whose outcomes are behavioral. It also implies that intensity-only measurement, still the dominant practice in this literature, is likely to under-detect clinically meaningful pain change in SUD treatment trials and observational studies. These observations emerge from a scoping rather than systematic review and were not subjected to meta-analytic comparison. Nonetheless, the convergence of pattern, mechanism, and clinical relevance is sufficient to elevate interference from a descriptive subdomain to a candidate measurement priority for the field.

### Validation gaps and SUD-specific psychometrics

A key finding of this review is the limited reporting of SUD-specific psychometric evaluation of pain measures. Fewer than one in six studies (14.6%) reported any psychometric data in SUD samples, and most of this evidence was confined to the BPI in OUD populations. For other commonly used tools, including the NRS, VAS, SF-36 pain components, and study-specific questionnaires, SUD-specific reliability and validity were rarely addressed, particularly in non-opioid substance use populations. In many cases, measures were adopted from general chronic pain or medical populations without explicit evaluation of their performance in the context of SUD.

This validation gap is consequential. There are concrete reasons to expect that the measurement properties of pain instruments may differ in SUD populations. We highlight three testable hypotheses for future psychometric work. First, state-dependent variability: item endorsement, factor structure, and responsiveness may all change across withdrawal, early stabilization, and sustained treatment phases, because the underlying pain-generating processes described in the Introduction shift across these states [21–23]. Second, construct overlap among pain, craving, and negative affect: in SUD populations these constructs are more closely coupled than in general chronic pain samples, which may inflate item-item correlations, alter factor structure, and reduce discriminant validity for pain-specific items. Third, differences in responsiveness to clinically meaningful change, particularly for items keyed to mood and activity, which may shift for non-pain reasons during SUD treatment. Concrete next steps include longitudinal measurement invariance testing across treatment phase, bifactor models that partition pain-specific variance from general distress, and minimal important difference estimation anchored to SUD-relevant outcomes such as relapse, retention, and functional recovery.

### Heterogeneity of pain-generating processes and interpretation in OUD

Assessing pain in people who use substances including opioids presents additional complexities, but framing those complexities as a problem of “separating pain from withdrawal” is conceptually imprecise and risks delegitimizing valid patient-reported experience. Under the IASP definition, withdrawal-related somatic distress is itself pain. The interpretive challenge is that a given pain rating in OUD may reflect a shifting mixture of nociceptive, nociplastic, and withdrawal-related contributors, alongside affective and cognitive processes. Their neural substrates overlap substantially. This is particularly true for the overlap between hyperkatifeia and the central sensitization characteristic of nociplastic pain [17, 21–23, 138]. Several studies in our sample acknowledged this heterogeneity, but few systematically assessed or reported withdrawal status, treatment phase, or timing of pain measurement relative to dosing.

This has direct implications for both clinical practice and research. Without explicit assessment of treatment state and the timing of measurement relative to dosing, clinicians and investigators may misinterpret elevated pain scores. They may attribute pain to the wrong contributor and adjust treatment inappropriately or alternatively dismiss valid pain reports as “just withdrawal.” Future studies should incorporate standardized measures of withdrawal and the timing of assessment relative to dosing alongside pain measurement. Psychometric work should examine how pain scales behave across withdrawal, stabilization, and sustained-treatment states. The goal is not to label individual pain reports as “real” versus “withdrawal” but to support measurement that captures the dominant contributor or contributors at the moment of assessment.

### From descriptive mapping to pragmatic assessment strategies

Our findings support efforts to move beyond intensity-only assessment toward brief, multidimensional approaches that capture domains more closely tied to functioning and SUD outcomes. The observation that pain interference more often showed stronger associations with substance use outcomes than intensity alone in studies that directly compared these domains suggests that interference and functioning are clinically important targets for measurement. At the same time, many SUD treatment settings face substantial time and resource constraints, which limits the feasibility of lengthy instruments.

We propose a pragmatic, evidence-informed minimum set for pain assessment in SUD intake and follow-up. The Graded Chronic Pain Scale-Revised (GCPS-R) provides a brief assessment of pain chronicity and impact, including classification of high-impact chronic pain, and incorporates the three multi-dimensional PEG items [133]. The PEG scale, derived from the BPI, includes three items assessing pain intensity(P) and interference with enjoyment(E) of life and general activity(G). These items cover the interference domains most consistently linked to SUD outcomes in this review [64, 132]. A brief body map, of the kind already widely used in chronic pain research [11, 27], adds anatomical information relevant to mechanism and treatment planning. For example, body map patterns can help distinguish nociplastic from nociceptive contributions [11, 21]. Together, the combination of GCPS-R and a body map is a multi-item minimum set that aligns with HEAL pain CDE domains, captures intensity and interference, supports identification of high-impact chronic pain, and is feasible for SUD intake and follow-up workflows. We present this as a candidate minimum set emerging from the patterns observed in this review. Prospective validation in diverse SUD treatment settings is needed before it should be treated as a standard.

### Implementation considerations

Implementation guidance for pain assessment in SUD settings was variably reported in the included studies. A minority described specific assessment frequencies or integration into routine intake procedures, and even fewer addressed how pain assessment results informed treatment decisions, referrals, or interdisciplinary care pathways. In practice, SUD programs operate within constrained workflows and may have limited access to pain specialists. It is therefore important to embed pain assessment in a way that is efficient and actionable. Brief multidimensional tools that can be incorporated into standard intake or periodic review processes, with clear protocols for follow-up assessment or referral, may support more consistent identification and management of pain in SUD populations [34, 139]. 

### Research priorities

Several research priorities emerge from this review. First, targeted psychometric evaluation of commonly used pain measures, and of brief multidimensional combinations such as GCPS-R and a body map, is needed in SUD populations across different substances and treatment settings. These studies should examine reliability, construct validity, responsiveness, and measurement invariance, including the impact of treatment phase, withdrawal status, and co-occurring mental health conditions. Second, longitudinal research that incorporates repeated pain assessments at clinically meaningful time points, including initiation of treatment, early recovery, and sustained remission, would clarify how pain trajectories relate to substance use outcomes and whether changes in specific domains, especially interference, predict relapse or retention [64, 89]. 

Third, technology-enhanced approaches, including ecological momentary assessment and digital monitoring tools, may offer opportunities to capture real-time fluctuations in pain and their relationship to substance use behaviors [89]. Finally, implementation science studies that evaluate strategies to integrate brief multidimensional pain assessment into SUD treatment workflows are needed. These studies should examine clinician training, decision support, and interdisciplinary care models, and they should move beyond measurement description toward sustainable practice change [140, 141]. 

### Strengths and limitations

This review has several strengths. We used a comprehensive, research librarian-informed search strategy across multiple databases. We followed PRISMA-ScR and PRISMA-S guidance and registered the protocol prospectively [24, 25]. Study selection and data extraction were conducted by multiple independent reviewers, with discrepancies resolved through consensus. We systematically mapped pain measures to a multidimensional framework based on NIH HEAL pain CDE domains. We also summarized methodological quality using a modified Newcastle-Ottawa Scale adapted for pain assessment in SUD research, providing context for interpreting the evidence base.

Several limitations should be noted. First, the included studies spanned 1990 to 2025, a period characterized by substantial changes in pain management, opioid prescribing practices, and recognition of the opioid crisis, as well as the introduction of the HEAL initiative in 2018 [1, 28, 29]. The 1990 lower bound was chosen to bracket the introduction of the first generation of validated multidimensional pain measures, but it also means that many included studies were not designed with current multidimensional frameworks in mind. HEAL domains were therefore applied retrospectively as an organizing structure. Domain coverage estimates should be interpreted as descriptive rather than as evidence of non-adherence to contemporary standards. Retrospective mapping of original measures to HEAL domains also involves judgment about item content and can misclassify multi-purpose items, for example items intended as intensity probes that also touch on interference. Coverage estimates should therefore be read as approximate. Second, heterogeneity in study designs, populations, settings, and measures precluded meta-analysis and limited our ability to formally compare effect sizes or predictive performance of different tools.

Third, SUD-specific psychometric data were often sparse and inconsistently reported. Our estimates of validation frequency may therefore underestimate informal psychometric work that was not fully described in manuscripts. Fourth, we restricted inclusion to English-language publications or publications with available English translations, which may limit generalizability to non-English-speaking contexts. Fifth, although we extracted information on exploratory pain-substance use outcome associations, these were not primary predefined outcomes of the scoping review and were reported qualitatively. Causal inferences cannot be drawn from these patterns. Finally, as with all scoping reviews, the emphasis on breadth rather than depth means that specific tools or subpopulations may warrant more detailed examination than was possible here.

## Conclusions

Systematic gaps exist in how pain is measured in SUD research. These include frequent reliance on intensity-only assessments, limited SUD-specific psychometric evaluation of pain measures, and incomplete coverage of multidimensional pain domains. The gaps are particularly notable in non-opioid substance use populations and in domains such as interference, functioning, sleep, and treatment satisfaction. These domains are closely tied to everyday functioning and treatment engagement. Pain interference more consistently tracks substance use outcomes than intensity alone in the studies that directly compared the two, supporting its elevation from a descriptive subdomain to a candidate measurement priority.

Adopting brief, multidimensional assessment strategies, specifically the combination of GCPS-R and a body map, offers a pragmatic, evidence-informed minimum set to improve pain measurement in SUD research and clinical care. Prospective validation is needed, along with attention to treatment state, stigma, and equity considerations [64, 132, 133]. Coordinated efforts in psychometric research, longitudinal study design, technology-enabled assessment, and implementation science will be needed to ensure that pain measurement in SUD populations is both scientifically robust and clinically meaningful. Better measurement of pain in people with SUD can support more accurate treatment planning, reduce suffering, and improve addiction care.

## Supplementary information

Below is the link to the electronic supplementary material.

Supplementary Material 1

## Data Availability

Data generated and analyzed during the current study are available from the corresponding author on reasonable request.

## Abbreviations

BPI: Brief Pain Inventory
CDE: Common Data Elements
GCPS-R: Graded Chronic Pain Scale–Revised
HEAL: Helping to End Addiction Long-term
MeSH: Medical Subject Headings
NIH: National Institutes of Health
NRS: Numeric Rating Scale
OUD: Opioid Use Disorder
PEG: Pain, Enjoyment of life, General activity
PRISMA-ScR: Preferred Reporting Items for Systematic Reviews and Meta-Analyses extension for Scoping Reviews
PRISMA-S: PRISMA Search extension
SUD: Substance Use Disorder
VAS: Visual Analog Scale
