# Supplementary material for: Pain assessment tools in patients with substance use disorders: a systematic scoping review
Source: Addict Sci Clin Pract. 2026 Jun 11;21:66. doi: 10.1186/s13722-026-00686-y (PMC13563765; doi:10.1186/s13722-026-00686-y)
Supplement: Supplementary file 1 [file 13722_2026_686_MOESM1_ESM.docx]

**Supplement:**

**Pain Assessment Measures Used in Populations with Substance Use Disorder: A Systematic Scoping Review Protocol**

Background Substance use disorder (SUD), particularly opioid use disorder (OUD), presents unique challenges in pain assessment and management. The complex interaction between chronic pain, acute pain episodes, and substance use creates a need for validated and reliable pain assessment tools. However, the landscape of pain measures used in this population remains unclear, potentially leading to suboptimal assessment and treatment decisions.

Review Question and Objectives Primary Question What pain measures (screening tools and outcome measures) are currently being used to assess pain in adult populations with substance use disorder, with particular attention to opioid use disorder?

Objectives

1. To identify and map the range of pain measures used in research and clinical practice with SUD populations
2. To examine the contexts and settings in which these measures are being implemented
3. To identify any validation studies of pain measures specifically for SUD populations
4. To highlight gaps in current pain assessment approaches for this population

Inclusion Criteria Participants

- Adults (≥18 years) with diagnosed substance use disorder (any substance)
- Subgroup analysis will focus on opioid use disorder
- Both active use and recovery phases will be included

Concept

- Pain assessment tools, measures, and outcomes
- Including but not limited to:
  - Standardized pain scales
  - Patient-reported outcome measures
  - Clinician-administered assessments
  - Behavioral pain measures
  - Multidimensional pain assessments

Context

- Any healthcare or research setting
- No geographical limitations
- Any time period from 1990-2025
- Published in English language or available translation

Types of Sources

- Peer-reviewed journal articles
- Clinical practice guidelines
- Systematic reviews
- Validation studies
- Clinical trials
- Observational studies
- Qualitative studies exploring pain assessment

Search Strategy Electronic Databases

- MEDLINE (via PubMed)
- EMBASE
- PsycINFO
- CINAHL
- Cochrane Library
- OVID

Search Terms Core Concepts

1. Substance Use Disorder:
   - "substance use disorder" OR "drug abuse" OR "drug dependence" OR "substance abuse" OR "substance dependence" OR "opioid use disorder" OR "addiction"
2. Pain Assessment:
   - "pain measurement" OR "pain assessment" OR "pain scale" OR "pain questionnaire" OR "pain outcome" OR "pain screening" OR ("PROs" AND "pain") OR ("Patient reported outcomes" AND "pain")
3. Measurement Properties:
   - "psychometric" OR "validity" OR "reliability" OR "responsiveness" OR "clinimetric" OR "measurement properties"

**Specific search terms by database**

1. PubMed Search String: (("Pain Measurement"[Mesh] OR "Pain Assessment"[Mesh] OR pain measure*[tiab] OR pain evaluat*[tiab] OR pain scale*[tiab] OR pain rating*[tiab] OR pain inventor*[tiab] OR pain questionnaire*[tiab] OR pain assessment tool*[tiab]) AND ("Opioid-Related Disorders"[Mesh] OR "Substance-Related Disorders"[Mesh] OR opioid use disorder*[tiab] OR opioid addiction*[tiab] OR opioid dependen*[tiab] OR opioid abuse*[tiab] OR substance use disorder*[tiab] OR prescription opioid misuse*[tiab]))
2. Ovid MEDLINE(R) ALL <1946 to February 07, 2025>: 1)exp Pain Measurement/ or exp Pain Assessment/99393; 2)limit 1 to (english language and humans)81758: 3)(pain adj3 (measure* or evaluat* or scale* or rating* or inventor* or questionnaire* or assessment tool*)).ti,ab,kf.82584; 4)	1 or 3. 153803; 5) 2 and 4 81758; 6) Substance-Related Disorders/ or Narcotic-Related Disorders/ or Opioid-Related Disorders/ 132591; 7) 5 and 6 526
3. Embase Search String (Ovid syntax): (exp AND ('pain'/exp OR pain) AND ('assessment'/exp OR assessment) OR exp) AND ('pain'/exp OR pain) AND ('measurement'/exp OR measurement) AND (exp Opiate Addiction/ OR exp Substance Abuse/ OR (opioid adj3 (use disorder* or addiction* or dependen* or abuse*)) OR substance use disorder* OR prescription opioid misuse*)
4. CINAHL: (import into Covidence as an XML file: ((MH "Pain Measurement+") OR (MH "Pain Assessment") OR TI ( pain N3 (measure* OR evaluat* OR scale* OR rating* OR inventor* OR questionnaire* OR "assessment tool*") ) OR AB ( pain N3 (measure* OR evaluat* OR scale* OR rating* OR inventor* OR questionnaire* OR "assessment tool*") )) AND ((MH "Substance Use Disorders+") OR (MH "Opioid-Related Disorders+") OR TI ( (opioid* N3 ("use disorder*" OR addiction* OR dependen* OR abuse*)) OR "substance use disorder*" OR "prescription opioid misuse*" ) OR AB ( (opioid* N3 ("use disorder*" OR addiction* OR dependen* OR abuse*)) OR "substance use disorder*" OR "prescription opioid misuse*" ))
5. APA PSYCHINFO - found in EBSCOHost database (same as CINAHL): ((MH "Pain Measurement+") OR (MH "Pain Assessment") OR TI ( pain N3 (measure* OR evaluat* OR scale* OR rating* OR inventor* OR questionnaire* OR "assessment tool*") ) OR AB ( pain N3 (measure* OR evaluat* OR scale* OR rating* OR inventor* OR questionnaire* OR "assessment tool*") )) AND ((MH "Substance Use Disorders+") OR (MH "Opioid-Related Disorders+") OR TI ( (opioid* N3 ("use disorder*" OR addiction* OR dependen* OR abuse*)) OR "substance use disorder*" OR "prescription opioid misuse*" ) OR AB ( (opioid* N3 ("use disorder*" OR addiction* OR dependen* OR abuse*)) OR "substance use disorder*" OR "prescription opioid misuse*" ))

Grey Literature Sources

- Clinical trial registries
- Pre-print servers (e.g., MedRxiv)
- Professional organization websites
- Conference proceedings
- Dissertations and theses

Study Selection Screening Process

1. Title and abstract screening by at least two independent reviewers
2. Full-text review of selected articles by at least two independent reviewers
3. Conflicts resolved through discussion or third reviewer arbitration
4. Documentation of selection process using PRISMA-ScR flow diagram
5. Literature search process using PRISMA-S

Data Extraction Data will be extracted using a standardized form including:

- Study characteristics (design, setting, population)
- Pain measure characteristics
- Implementation context
- Psychometric properties (if reported)
- Clinical utility information
- Limitations and challenges reported

Analysis and Presentation Data Synthesis

- Narrative synthesis of findings
- Modified NOS Scale
- Mapping of pain measures across different contexts and populations
- Analysis of measure characteristics and implementation factors
- Identification of knowledge gaps and research needs

Results Presentation

- Tables summarizing identified pain measures
- Visual mapping of measure usage across contexts
- Quality assessment of validation evidence
- Gaps analysis matrix

Timeline

- Protocol Registration: [January, 2025]
- Literature Search: [February, 2025]
- Screening and Selection: [February, 2025]
- Data Extraction: [Feb-March, 2025]
- Analysis and Synthesis: [Mar-April, 2025]
- Manuscript Preparation: [June, 2025]

Initial Team Members: Meredith Adams MD, PI; Screening, review, extraction, analysis and manuscript preparation Robert Hurley MD/PhD, Co-I; Screening, review, extraction, analysis and manuscript preparation Cara McDonnell MPH, Program Manager; Screening, review, extraction Clinical research technician team; Screening, review, extraction

Full Team Members: Meredith Adams MD, PI; Screening, review, extraction, analysis and manuscript preparation - Robert Hurley MD/PhD, Co-I; Screening, review, extraction, analysis and manuscript preparation - Cara McDonnell MPH, Program Manager; Screening, review, extraction Clinical research technician team; Screening, review, extraction - Elizabeth Shilling PhD; Analysis and manuscript preparation – Steven Abriola MD; Analysis and manuscript preparation – Jennifer Oliver DO MPH; Analysis and manuscript preparation

Funding NIH-NIDA - IMPOWR, MIRHIQL

Registration This protocol was be registered with the Open Science Framework (OSF) prior to initiating the review process.

Dissemination Plans

- Submission to peer-reviewed journal
- Conference presentations
- Stakeholder engagement activities
- Open access publication when possible
